# Plasmonic colloidal pastes for surface-enhanced Raman spectroscopy (SERS) of historical felt-tip pens†

**DOI:** 10.1039/c7ra13464a

**Published:** 2018-02-22

**Authors:** Daniela Saviello, Abeer Alyami, Maddalena Trabace, Rodorico Giorgi, Piero Baglioni, Antonio Mirabile, Daniela Iacopino

**Affiliations:** Tyndall National Institute, University College Cork Dyke Parade Cork Ireland daniela.iacopino@tyndall.ie; Department of Chemistry, CSGI, University of Florence Italy; Mirabile 11 Rue de Bellefond 75009 Paris 09 France

## Abstract

Surface-enhanced Raman spectroscopy (SERS) has been identified as a suitable technique for the analysis of colorants in works of art. Herein, the application of SERS to the identification of dye compositions in historical felt-tip pens is reported, which is of paramount importance for the development of appropriate conservation protocols for historical drawings. In this study, three pens (pink, green, and blue colors) belonging to the film director Federico Fellini were analyzed. SERS measurements were performed directly on the pen lines drawn on a commercial paper by the deposition of Ag colloidal pastes, which allowed fast *in situ* dye identification without the need for extraction or hydrolysis treatments. Eosin Y was identified as the only dye present in the pink pen ink, whereas erioglaucine was found to be the main dye component in green and blue pen inks. SERS also resulted in highly efficient identification of the individual dyes erioglaucine, crystal violet, and rhodamine present as a mixture in the blue pen ink. The high SERS sensitivity was ascribed to the plasmonic effects and efficient quenching of the fluorescence interference of dyes. A comparison with contemporary pen inks highlighted minor differences in the chemical composition. These results prove that SERS can be used as a fast and sensitive analytical tool for ink analysis that provides invaluable support for the general assessment of the date, provenance, and originality of the historical drawings as well as for the development of preventive conservation protocols.

## Abstract 2

SERS analysis performed with silver nanopastes allowed *in situ* identification of dye compositions of the historical felt-tip pens used by Federico Fellini.

## Graphical abstract

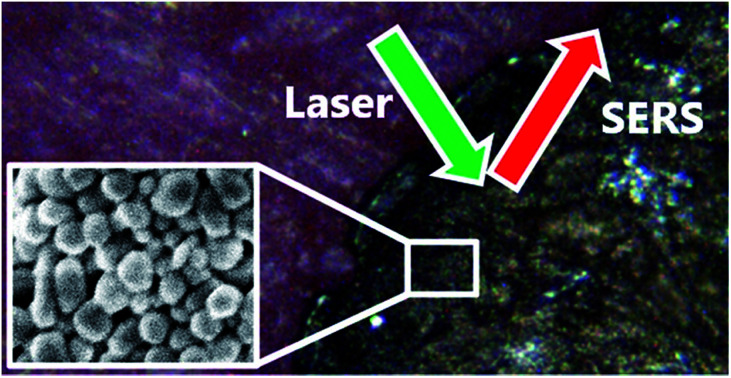

## Introduction

Felt-tip pens were introduced in the market in 1953 by Rosenthal and became commonly used by many influential artists who left a large heritage of artistic productions today hosted by museums all over the world. Because of the well-known sensitivity of the inks to light, especially in the UV part of the spectrum, it is currently not known how these artworks will withstand the passage of time.^1,2^ In this context, identification of dyes constituting felt-tip pen ink mixtures is highly important for the (i) precise assessment of vulnerability, (ii) design of intervention protocols, and (iii) creation of ideal housing and storage conditions for historical drawings and writing materials. In addition, the elucidation of ink ID is relevant for the assessment of artwork date, provenance, and originality.

Interestingly, very few studies on the chemical composition of felt-tip pen have been carried out to date. Sodo *et al.*^3^ used Raman spectroscopy to analyze historical drawings, but found limitations such as low sensitivity, fluorescence interference of constituent artificial dyes and paper matrices, and inability to conclusively identify individual dyes in ink mixtures. Izzo *et al.*^4^ and Germinario *et al.*^5^ performed comprehensive analyses of felt-tip pen brands and colors. However, both studies employed multi-analytical approaches based on the use of high-cost instrumentation; moreover, these approaches were generally destructive and required relatively large amounts of sample. These approaches are invaluable for constituting reference artificial dye databases equivalent to the already established databases for organic colorants. However, they are not suitable for the analysis of historical drawings and writing materials, where the use of non-invasive and sensitive analytical techniques is mandatory to ensure the integrity of analyzed objects.

Over the past 10 years, SERS has become the diagnostic tool of choice for the identification of colorants in works of art.^6–8^ The SERS effect entails enhancement of the Raman scattering signal of an analyte spatially confined within the electromagnetic field generated upon excitation of the localized surface plasmon resonance (LSPR) of a nanostructured noble metal surface.^9^ Nowadays, it is widely accepted that SERS enhancement arises from two contributions: chemical effects (CM) and electromagnetic effects (EM).^10–13^ CM effects contribute 0 to 10^2^ of the total enhancement and result from charge transfer mechanisms between analyte molecules and the metal substrate.^14,15^ EM enhancements contribute 10^4^ to 10^12^ of the total enhancement and are due to the collective excitation of metal substrate surface plasmons by incident light.^16,17^ Due to these reported high signal enhancements over normal Raman signals, SERS is particularly amenable to the investigation of artistic materials where mass-limited samples are often available, and *in situ* applications and local identification of selective analytes are often required.^18,19^ Furthermore, SERS has sensibly expanded the range of measurable materials due to efficient quenching of the molecular fluorescence interferences of natural and artificial dyes.^20^ As a result, many authors have reported successful investigations of lakes and dyestuffs in archaeological textile fibers^6,21^ and paper and woodblock prints^22^ and the identification of dye contents in pastel colors,^7^ painting samples,^23–25^ and watercolor pigments.^26^ The ability to identify individual dyes within several dye mixtures was also reported by Whitney *et al.* who used silver film over nanospheres (AgFONs) as SERS substrates for the identification of several artists' red dye mixtures.^27^ SERS also provided an efficient alternative to routinely used chromatographic analysis for the identification of dyes in commercial pens of forensic interest such as blue and black ballpoint pens, for which, the chemical composition of ink is difficult to access due to trademark protection.^28–30^ In parallel, many efforts have also been devoted to the fabrication of robust, stable, reproducible, and plasmonically tailorable SERS substrates to enhance the sensitivity and reproducibility.^31,32^ However, Ag colloidal solutions remain the most used SERS probes due to ease of their preparation, low cost of their production, and their relatively good chemical stability.

However, one of the major drawbacks associated with the application of SERS to the analysis of colorants is the combined use of harsh extraction methods. Hydrolysis procedures are in fact often required to separate colorants from their matrices and promote their adsorption on Ag colloids. However, application of these procedures often results in disruption of the host material and formation of interference degradation products.^33^ In the last six years, Ag colloidal pastes were proposed as an alternative for *in situ* non-hydrolysis SERS measurements.^6,19,34^ This approach consisted of the deposition of concentrated colloidal solution droplets on untreated analytical samples and was particularly effective for the characterization of historical textile fibers. However, limitations such as poor spatial resolution, difficulty in achieving homogeneous sample coverage, and consequently poor reproducibility were also identified.^34^

Herein, we present the first study in which SERS is applied for the analysis of historical felt-tip pens belonging to the film director Federico Fellini. In this study, three pens were analyzed directly on paper matrices by the *in situ* deposition of Ag-concentrated colloidal solutions (pastes). Comparison between Raman and SERS measurements revealed that the use of plasmonic pastes, which were wrapped around paper-colored fibers creating large areas of high-density Ag nanoparticles, was key for the achievement of enhanced SERS *in situ* signals relative to Raman signals. The high specificity of SERS allowed identification of the main dyes for all the analyzed inks and discrimination between individual dye components in ink mixtures. Moreover, SERS proved highly efficient in identifying subtle compositional changes between differently dated inks. These characteristics are highly sought for the analysis of real artworks. This study represents yet another example of the superior sensitivity of SERS and its suitability for art conservation diagnostics. In addition, the successful application of plasmonic pastes stressed the importance of efficient plasmonic material engineering in further enhancing the SERS sensitivity and expanding its applicability within the field of art conservation and preservation.

## Results and discussion

Federico Fellini (1920–1993), recognized internationally as a film director and screen writer, drew extensively between the 1960s and 1990s often to capture on paper the characters of his films. Fellini has left a large patrimony of sketches, notes, and drawings that are today housed by the studio Fellini of Cinecittà (Rome, Italy) and the Fellini Foundation (Rimini, Italy). The present study focused on the identification of dye contents in felt-tip pens owned by Fellini. As some of the Fellini's drawings already showed signs of damage in the form of water stains and tape marks, the aim of this study was the generation of ESI† for the future development of conservation and intervention protocols towards the long-term preservation of Fellini's and other felt-tip pen-based historical drawings.

**Scheme 1:**
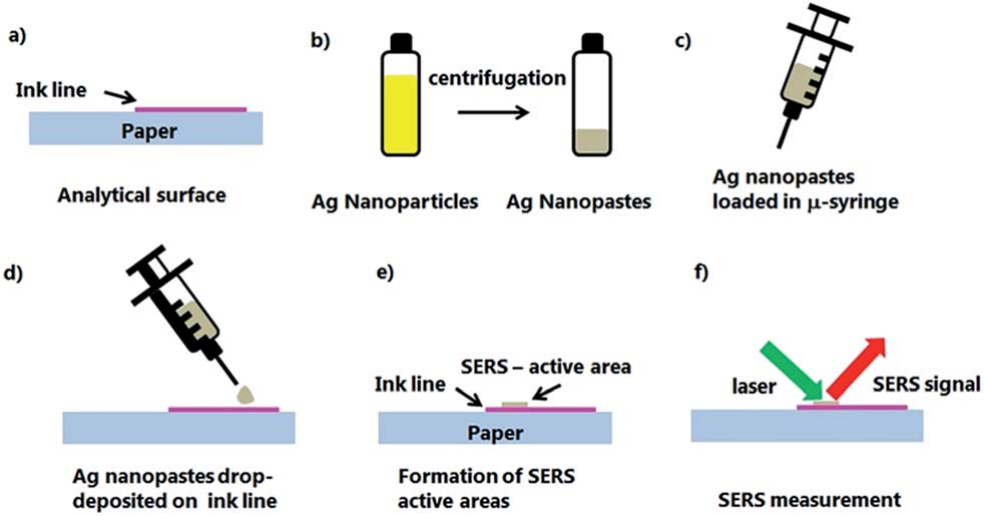
Schematic of sample preparation for *in situ* SERS measurements of historical drawings: (a) analytical sample constituted by ink coloured paper; (b) formation of nanopaste by centrifugation of Ag nanoparticle solutions; (c) loading of Ag nanopaste into a micro-syringe; (d) droplet deposition of Ag nanopaste on the analytical surface; (e) formation of SERS-active areas on the analytical surface; and (f) SERS measurement.

Scheme 1 depicts the stages followed to perform *in situ* analysis on Fellini's historical pens. Individual lines (*ca.* 2 cm long) were drawn on a commercial paper with three selected felt-tip pens: pink Tombow ABT 813, green Caran D'Ache Fibralo series 100, and blue Caran D'Ache Fibralo series 100 (Scheme 1a). The plasmonic paste was synthesized according to a method developed by Polavarapu *et al.*^35^ that consisted of reduction of the volume of Ag nanoparticles obtained by the classical method of Lee and Meisel by two orders of magnitude to obtain colloidal suspensions of high viscosity (Scheme 1b).^36^ SERS-active areas were obtained by depositing 3 μL droplets of the Ag colloidal paste directly on the paper-colored lines (Scheme 1c and d). SERS measurements were performed on the SERS active areas formed directly on the paper-deposited ink lines (Scheme 1e and f).

**Fig. 1:**
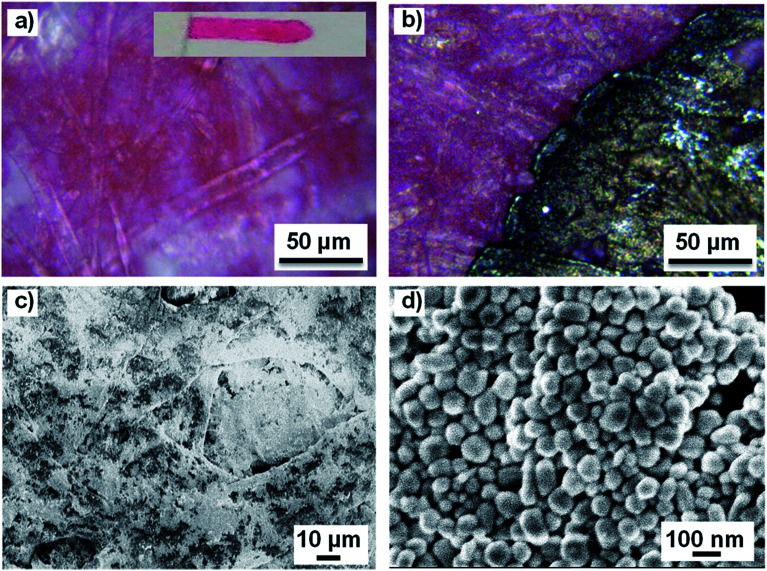
(a) Optical image of the pink Tombow ABT 813 felt-tip pen line written on a commercial paper. Inset: an image of the entire line. (b) Optical image of the pink line on a paper with deposited Ag plasmonic paste. (c) SEM image of the pink-colored paper with deposited Ag plasmonic paste. (d) High-magnification SEM image of the paper-deposited Ag colloidal paste showing the morphology of individual particles.

A representative image of the analyzed line is shown in Fig. 1a, displaying pink Tombow ABT 813 felt-tip pen color deposited on Fabriano paper fibers. The image of the entire line is shown in the inset. Fig. 1b shows an optical image of the pink Tombow colored line on paper with a deposited SERS-active area. Fast solvent evaporation associated with the high concentration of Ag colloids in the droplet resulted in the formation of well-confined and relatively uniform SERS-active areas. Concomitantly, the use of high-concentration plasmonic pastes prevented dissolution of the water-soluble felt-tip pens into the Ag colloidal solution and therefore allowed retention of a high concentration of analytical ink on the paper in direct contact with the plasmonic material (see Fig. S1† for comparison with diluted Ag colloidal solution deposition). The SEM image of the Ag colloidal paste deposited on a colored paper (Fig. 1c) shows dense and uniform nanoparticle coverage of multiple paper fibers and the consequent formation of large areas of hot-spot concentrations necessary for achieving sensitive SERS detection. Fig. 1d displays a high magnification SEM image of the deposited plasmonic paste showing individual spherical and rod-shaped particles with an average diameter of 100 ± 5 nm.

**Fig. 2:**
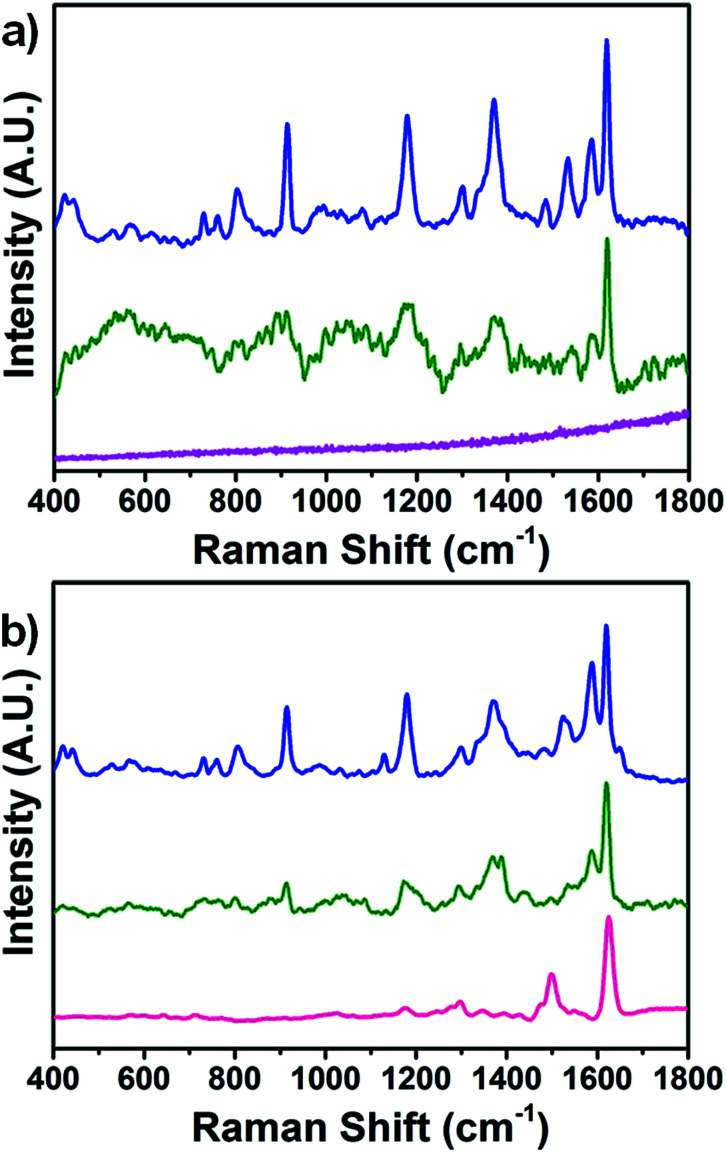
Raman (a) and SERS (b) spectra of lines written on paper with pink Tombow ABT 813 (pink line), green Caran D'Ache Fibralo series 100 (green line) and blue Caran D'Ache Fibralo series 100 (blue line).

Fig. 2a shows the normal Raman (NR) spectra of the three analyzed pen lines obtained using a 514 nm laser. The NR spectrum of the pink Tombow ink was featureless. This is not surprising as red-colored inks are characterized by strong absorption close to the selected excitation wavelength (514 nm); this often results in the concomitant generation of interference fluorescence emission and consequent masking of Raman signals.^22^ The green Caran D'Ache ink showed a low quality NR spectrum with distinctive peaks at 1622, 1587, and 1295 cm^−1^. The blue Caran D'Ache ink showed an NR spectrum of good quality with high intensity peaks. For this ink, the comparison of spectral features with NR spectra measured for 12 reference dyes allowed the preliminary identification of erioglaucine (acid blue 9, brilliant blue FCF (CI 42090)) from the peaks located at 1619–1587–1534 cm^−1^, 1490–1446 cm^−1^, 1430–1370 cm^−1^, 1177 cm^−1^, 915 cm^−1^, and 759 cm^−1^.^37^ Additional vibrational bands attributable to crystal violet (CI 42555) were observed at 1583, 1532, 1372, 1297, 1177, 913, 802, 439, and 422 cm^−1^.^38^ The generation of a high intensity NR spectrum for the blue ink was ascribed to decreased fluorescence interference associated with the shift of resonant absorbance to higher wavelengths (600–650 nm) than the 514 nm excitation wavelength. Fig. 2b shows the SERS spectra of the three analyzed pen lines obtained at the illumination wavelength 514 nm. All pens showed high intensity spectra with distinctive spectral features, which resulted in general enhancement as compared to the previously observed Raman features. The enhancement was particularly striking for the pink ink as it contrasted with its featureless Raman spectrum. Specifically, vibrational bands attributable to Eosin Y (CI 45380) were observed at 1624 cm^−1^, 1500 cm^−1^, 1296 cm^−1^, and 1176 cm^−1^.^39^ Modest enhancement and generation of additional diagnostic peaks as compared to that in the Raman spectrum was observed for the green ink, which allowed identification of erioglaucine as a blue component from the peaks located at 1619–1587–1534 cm^−1^, 1490 cm^−1^, 1370 cm^−1^, 1294 cm^−1^, 1177 cm^−1^, and 910 cm^−1^. The SERS spectrum of the blue ink was comparable in intensity to the Raman spectrum; this confirmed the presence of erioglaucine and crystal violet in the blue ink mixture.

**Fig. 3:**
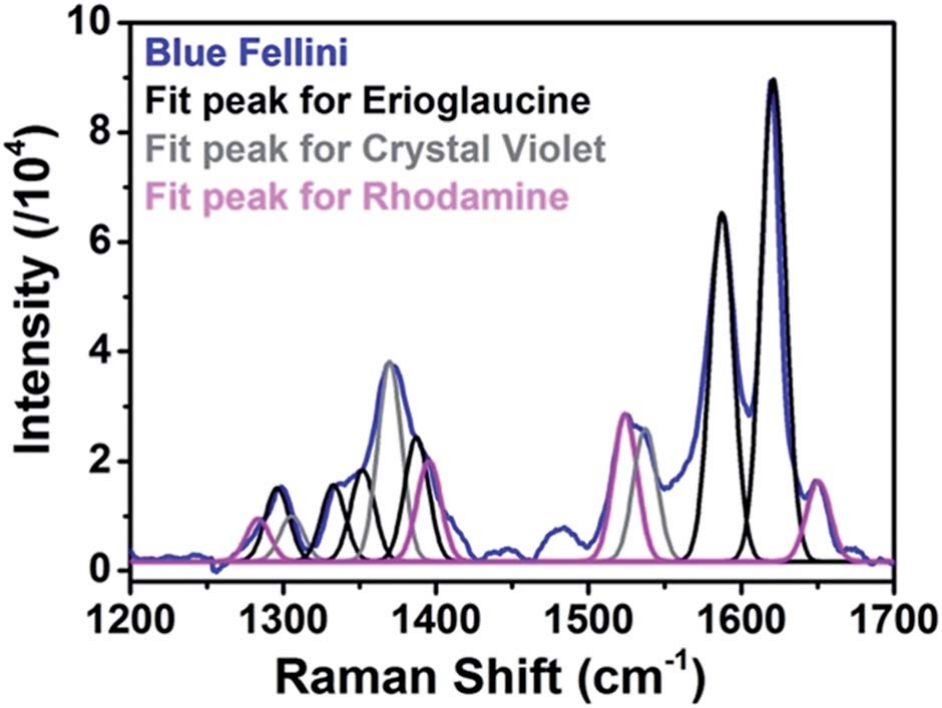
SERS spectrum of the blue Caran D'Ache felt-tip pen line (blue line) and Gaussian fits of identified dye components erioglaucine (black line), crystal violet (grey line), and rhodamine (pink line).

However, a closer observation of the blue ink SERS spectrum revealed the formation of a peak at 1650 cm^−1^ that was not observed in the Raman spectrum. Further deconvolution analysis (Gaussian mathematical fitting) (see Fig. 3) allowed association of the formation of the 1650 cm^−1^ peak with the presence of a red rhodamine component (either B of 6G) in the blue ink mixture only visible under SERS conditions. The capability of SERS for simultaneous and fast identification of dye mixtures is highly relevant for real artwork analysis where a combination of dyes is often used to achieve the desired hue. Details of peak positions and assignments of all the analyzed inks are reported in Table S1.†

**Fig. 4:**
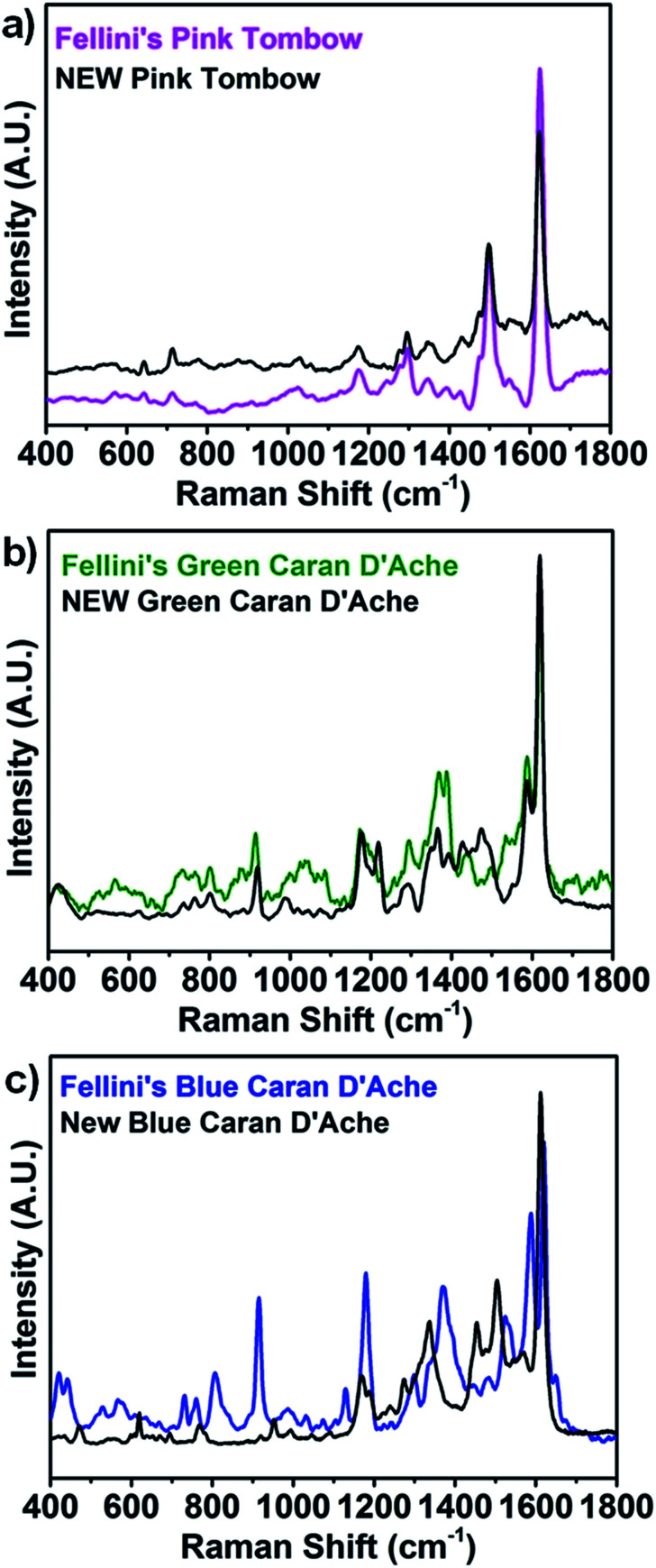
Comparison between the historical and contemporary commercial felt-tip pens. SERS spectra of (a) pink Tombow ABT 813 (pink line) and pink Tombow 755 (black line); (b) green Caran D'Ache Fibralo 100 (green line) and green Caran D'Ache 185 (black line); and (c) blue Caran D'Ache Fibralo 100 (blue line) and blue Caran D'Ache 185.

Finally, the ability of SERS to distinguish small compositional changes occurring between equivalent pens of different production years was assessed as relevant for dating, provenance, and authenticity attributions. Moreover, three contemporary felt-tip pens with colors and brands equivalent to the historical pens analyzed were purchased from local stores. Colored lines were drawn on a commercial paper, and SERS spectra were obtained under the conditions described for historical pen inks. SERS spectra of the contemporary pink Tombow 755 and green Caran D'Ache 185 inks (Fig. 4) show ink compositions equivalent to the historical pink and green inks that are indicative of the presence of Eosin Y and erioglaucine, respectively. Contemporary blue Caran D'Ache also showed dye composition equivalent to that of historical blue Caran D'Ache (erioglaucine and crystal violet). However, the contemporary blue ink red component was identified as Eosin Y, in contrast to rhodamine identified in the historical blue ink. It should be stressed that the enhancement provided by SERS is the key for the identification of structural differences between the abovementioned inks. In fact, equivalent comparison between Fellini and contemporary ink NR spectra (Fig. S2†) could only identify significantly different spectral features for the blue Caran D'Ache ink and was ineffective for pink and green inks.

The identification of dyes in the pen ink mixtures allowed further understanding of the origin of the observed enhancement of SERS signals as compared to that of the NR signals. The LSPR of the Ag colloidal solution obtained prior to solvent concentration and formation of paste was centered at 416 nm (see Fig. S3†). The formation of paste and its subsequent deposition on the paper surface contributed to significant red shift (478 nm, data not shown) of the LSPR towards the laser illumination wavelength such that all SERS measurements were performed close to plasmonic resonance conditions. This explains the general enhancement of the SERS spectra as compared to that of the NR spectra observed for all the analyzed inks. In the case of pink Tombow ink, Raman signals might have been further enhanced by illumination conditions in molecular absorbance resonance with Eosin Y (*λ*_max_ 516 nm in an aqueous solution, surface enhanced resonance Raman scattering conditions, SERRS). In this case, enhancement factors that are approximately the product of the enhancement factors of the non-resonant SERS substrate and the resonant Raman spectrum of the analyte were obtained.^22^ Molecular resonance conditions could also be responsible for the appearance of the Raman bands of the red dye components in the SERS spectra of the historical and contemporary blue Caran D'Ache inks.

To further understand the contributions of different factors in the observed enhancements, Au nanorod colloidal nanopastes with LSPR at 757 nm were drop-deposited on ink-colored papers. SERS spectra are obtained with the illumination wavelength 514 nm and reported in S4.† Pink Tombow Fellini was characterized by a featureless spectrum equivalent to the NR spectrum and strikingly different from the spectrum obtained with Ag nanopastes. The absence of enhancement in spite of SERRS experimental conditions would suggest that a plasmonic effect occurs with Ag nanopastes that is responsible for the strong enhancement observed herein. Appreciable SERS enhancement of green Fellini ink was not observed with Au nanopastes; this suggested that the modest enhancement observed with Ag nanopastes was due to a plasmonic effect. For the blue Fellini ink, negligible differences were observed between spectra obtained under NR, SERS_Ag nanopaste, and SERS_Au nanopaste conditions. The peak at 1650 cm^−1^ observed for Ag nanopastes was attributed to the presence of a red rhodamine component (and not observable with Au nanopastes) and could be an indication of some weak plasmonic effects taking place in the blue ink.

## Experimental

### Materials

Silver nitrate, trisodium citrate, ascorbic acid and the reference dyes Eosin Y, erioglaucine, and crystal violet were purchased from Sigma-Aldrich and used without further purification. All glassware was cleaned with aqua regia and deionized water prior to nanoparticle synthesis. Historical felt-tip pens belonging to the film director Federico Fellini were made available by the studio Fellini in Cinecitta' (Italy). Contemporary felt-tip pens were purchased from local stores.

### Synthesis of the Ag colloidal paste

The silver plasmonic paste was synthesized following the modified Lee and Meisel method reported by Polavarapu *et al.*^35^ Briefly, 4.5 mL of trisodium citrate solution (1.00 wt% in water) was added to 200 mL of boiling AgNO_3_ (42 mg) solution in water under vigorous stirring. The reaction mixture was kept boiling for one hour and then cooled down to room temperature. The obtained solution of Ag nanoparticles was centrifuged at 7000 rpm for 20 minutes and then re-dispersed in 2 mL of H_2_O to obtain the Ag paste (3 mg mL^−1^).

### Scanning electron microscopy (SEM)

Scanning electron microscopy images of the Ag colloids deposited on the SiO_2_ substrates were acquired using a field emission SEM (JSM-6700F, JEOL UK Ltd) operating at the beam voltage of 5 kV.

### Optical microscopy

White light optical microscopy images of the felt-tip pen lines on a commercial paper were acquired using an Axioskop II (Carl Zeiss Ltd.) microscope equipped with a halogen lamp and a charge-coupled detector camera (CCD; Coolsnap CF, Photometrics).

### Raman and SERS analysis

Raman and SERS spectra were obtained at 514 nm using a Renishaw inVia Raman system with a helium–neon laser excitation source. The laser beam was focused on the sample through a Leica 20× objective with 0.4 NA. The power measured at the sampling level was in the range of 0.01–2 mW. Acquisition time was 10 s. Raman spectra of the felt-tip pens were obtained directly from felt-tip pen-colored lines on a commercial paper. To obtain the SERS spectra, 5 μL of Ag plasmonic paste was deposited on colored lines on the paper and left to dry overnight in the dark prior to analysis. Reference dyes were analyzed as a powder deposited on glass slides.

## Conclusions

In conclusion, this study presents the first application of SERS to the analysis of historical felt-tip pens. SERS measurements *in situ* were enabled by the use of plasmonic paste, which was deposited uniformly on pen-colored paper fibers, allowing sensitive detection. Compared to the standard colloidal solutions used for the analysis of historical dyes and pigments, the high nanoparticle concentration of the developed pastes enabled efficient *in situ* analysis of water-soluble pen inks. Compared to other Ag-based SERS platforms, such as AgFONs, commonly used in art conservation applications, plasmonic pastes offered simplicity and versatility of analysis and allowed circumventing of extraction steps. For all the pen inks analyzed, the higher sensitivity of SERS was ascribed mainly to plasmonic effects. However, other effects, such as effective quenching of fluorescence and measurements under SERRS conditions, could have contributed to the observed enhancements, especially those of the pink ink. The generation of high sensitivity SERS signals is particularly relevant for art conservation where extremely small amounts of material are available for analysis. Moreover, the possibility of *in situ* analysis, which allows circumventing invasive and time consuming extraction and hydrolysis steps, has the potential to sensibly widen the range of analyzed materials. The demonstrated capability of SERS to identify individual dyes in mixtures as well as the ability to discern changes in composition between differently dated inks further expand the use of SERS in art conservation to novel areas such as dating and attribution of originality and provenance. Finally, the data presented herein confirm the applicability of SERS as a valuable tool for the analysis of real art objects and as a support for the development of conservation or treatment protocols tailored to the long-term preservation of historical drawings.

## Conflicts of interest

There are no conflicts of interest to declare.

## Supplementary Material

RA-008-C7RA13464A-s001

## References

[cit1] Gherandi E., Degano I., Colombini M. P., Mazurek J., Schilling M., Khanjian H. (2015). Dyes Pigm..

[cit2] Papliaka Z. E., Andrikopoulos K. S., Varella E. A. (2010). J. Cult. Herit..

[cit3] Sodo A., Bicchieri M., Guiso M., Ricci M. A., Ricci G. (2012). J. Raman Spectrosc..

[cit4] Izzo F. C., Vitale V., Fabbro C., Van Keulen H. (2016). Microchem. J..

[cit5] Germinario G., Garrappa S., D'Ambrosio V., van der Werf I. D., Sabbatini L. (2018). Anal. Bioanal. Chem..

[cit6] Roh J. Y., Matecki M. K., Svoboda S. A., Wusthotlz K. L. (2016). Anal. Chem..

[cit7] Brosseau C. L., Rayner K. S., Casadio F., Grywacz C. M., Van Duyne R. P. (2009). Anal. Chem..

[cit8] Brosseau C. L., Gambardella A., Casadio F., Grywacz C. M., Wouters J., Van Duyne R. P. (2009). Anal. Chem..

[cit9] Jeanmaire D. L., Van Duyne R. P. (1977). J. Electroanal. Chem..

[cit10] Moskovits M. (1985). Rev. Mod. Phys..

[cit11] Moskovits M. (2005). J. Raman Spectrosc..

[cit12] Valley N., Greeneltetch N., Van Duyne R. P., Schatz G. C. (2013). J. Phys. Chem. Lett..

[cit13] Schatz G. C. (1984). Acc. Chem. Res..

[cit14] Kneipp K., Wang Y., Kneipp H., Perelman L. T., Itzkan I., Dasari R. R., Feld M. S. (1997). Phys. Rev. Lett..

[cit15] Fromm D. P. (2006). J. Chem. Phys..

[cit16] Qin L., Zou S., Xue C., Atkinson A., Schatz G. C., Mirkin C. A. (2006). PNAS.

[cit17] Blaber M. G., Schatz G. C. (2011). Chem. Commun..

[cit18] Pozzi F., Lombardi J. R., Bruni S., Leona M. (2012). Anal. Chem..

[cit19] Wustholz K. L., Brosseau C. L., Casadio F., Van Duyne R. P. (2009). Phys. Chem. Chem. Phys..

[cit20] Pozzi F., Leona M. (2016). J. Raman Spectrosc..

[cit21] Whitney A. V., Van Duyne R. P., Casadio F. (2006). J. Raman Spectrosc..

[cit22] Cesaratto A., Lombardi J. R., Leona M. (2017). J. Raman Spectrosc..

[cit23] Leona M. (2009). Proc. Natl. Acad. Sci. U. S. A..

[cit24] Pozzi F., Porcinai S., Lombardi J. R., Leona M. (2013). Anal. Methods.

[cit25] Zaffino A., Passaretti A., Poldi G., Fratelli M., Tibiletti A., Bestetti R., Saccani I., Guglielmi V., Bruni S. (2017). J. Cult. Herit..

[cit26] Pozzi F., Lombardi J. R., Leona M. (2013). Heritage Sci..

[cit27] Whitney A. V., Casadio F., Van Duyne R. P. (2007). Appl. Spectrosc..

[cit28] Geiman I., Leona M., Lombardi J. R. (2009). Forensic Sci..

[cit29] Seifar R. M., Verheul J. M., Ariese F., Brinkman U. A. T., Gooijer C. (2001). Analyst.

[cit30] Alyami A., Saviello D., McAuliffe M. A. P., Mirabile A., Lewis L., Iacopino D. (2017). Phys. Chem. Chem. Phys..

[cit31] Jurasekova Z., Domingo C., Garcia-Ramos J. V., Sanchez-Cortes S. (2008). J. Raman Spectrosc..

[cit32] Muniz-Miranda M., Gellini C., Giorgetti E. (2011). J. Phys. Chem. C.

[cit33] Leona M., Stenger J., Ferloni E. (2006). J. Raman Spectrosc..

[cit34] Idone A., Gulmini M., Henry A. I., Casadio F., Chang L., Apollonia L., Van Duyne R. P., Shah N. C. (2013). Analyst.

[cit35] Polavarapu L., Porta A. L., Novikov S. M., Coronado-Puchau M., Liz-Marzán L. M. (2014). Small.

[cit36] Lee P. C., Meisel D. (1983). Chem. Phys. Lett..

[cit37] Persaud I., Grossman W. E. L. (1993). J. Raman Spectrosc..

[cit38] Canamares M. V., Chenal C., Birke R. L., Lombardi J. R. (2008). J. Phys. Chem. C.

[cit39] Greeneltch N. G., Davis A. S., Valley N. A., Casadio F., Schatz G. C., Van Duyne R. P., Shah N. C. (2012). J. Phys. Chem. A.

