# Supplementary material for: Plasmonic colloidal pastes for surface-enhanced Raman spectroscopy (SERS) of historical felt-tip pens
Source: RSC Adv. 2018 Feb 22;8(15):8365–71. doi: 10.1039/c7ra13464a (PMC9078556; doi:10.1039/c7ra13464a)
Supplement: RA-008-C7RA13464A-s001 [file RA-008-C7RA13464A-s001.pdf]

Electronic Supplementary Material (ESI) for RSC Advances.
This journal is © The Royal Society of Chemistry 2018

Supporting Information

Plasmonic Colloidal Pastes for Surface Enhanced Raman Spectroscopy (SERS) of Historical Felt-tip Pens

Daniela Saviello(1), Abeer Alyami(1), Maddalena Trabace(2), Rodorico Giorgi(2), Piero Baglioni(2), Antonio Mirabile(3) and Daniela Iacopino(1)

(1) Tyndall National Institute, University College Cork, Dyke Parade, Cork, Ireland

(2) Department of Chemistry & CSGI, University of Florence, Italy

(3) Mirabile, 11 Rue de Bellefond, 75009 Paris 09, France

Deposition of diluted Ag colloidal solutions on colored paper

Figure S1 shows a photograph of a red line written on paper with commercial Stabilo felt tip pen. When diluted aqueous Ag colloidal solutions were deposited on the colored paper, the high water solubility of felt tip pens caused ink dissolution and substantial decrease of ink concentration in direct contact with the plasmonic surface. Therefore even deposition of multiple Ag colloidal droplets, as shown in Figure S1, was not effective for SERS measurements.

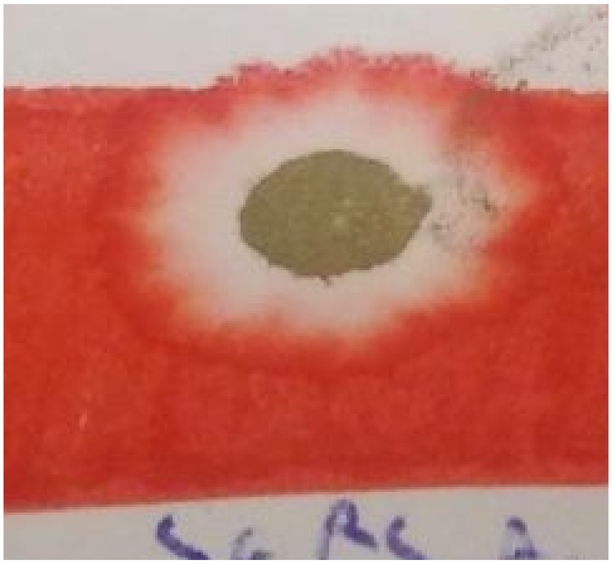

Figure S1. Photograph of commercial red felt tip line on paper with deposited Ag colloidal solution.

Comparison Fellini and contemporary pen ink NR spectra

In order to show the superiority of SERS for the identification of compositional differences, NR spectra of Fellini's pens (Figure S2a) were compared with NR spectra of equivalent color contemporary markers (Figure S2b). Figure S2 shows that for blue Caran D'Ache different spectral features were observed between the two analyszed blue markers, suggesting compositional difference. However, for green ink the resolution of NR spectra did not allow direct comparison between spectra. This also applied to analysis of pinks inks, which only produced featureless NR spectra.

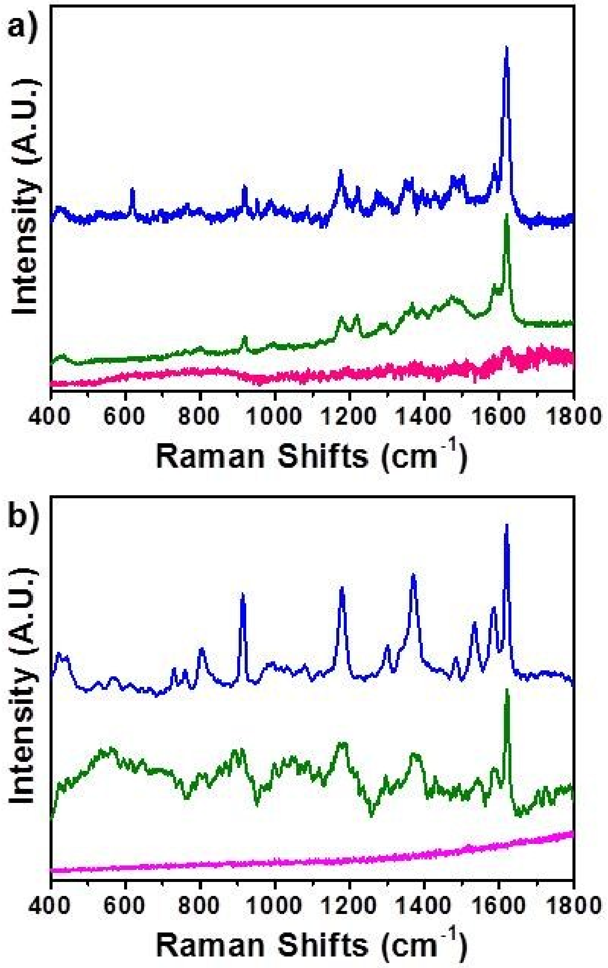

Figure S2. Comparison between historical and contemporary commercial felt tip pens. NR spectra of a) blue Caran D'Ache Fibralo series 100 (blue line), green Caran D'Ache Fibralo series 100 (green line) and pink Tombow ABT 813 (pink line); b) blue Caran D'Ache 185 (blue line). green Caran D'Ache 185 (green line) and pink Tombow (pink line).

Ag colloidal nanopastes

Ag colloidal pastes used for SERS analysis were synthesized according to a method developed by Polavaru et *al.*. Figure S3 shows a SEM image of the Ag colloids deposited on SiO_2 substrates, mostly constituted by spherical particles with an average size of 100 ± 5 nm. The colloidal solution was pale grey in color and showed a plasmonic peak centered at 416 nm (Figure S1 inset).

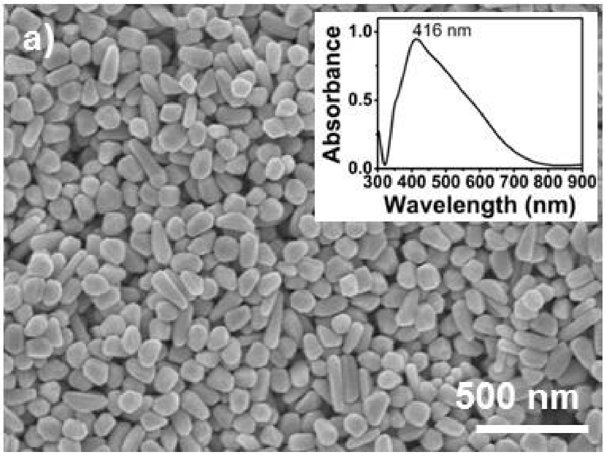

Figure S3. SEM images of Ag colloidal paste deposited on SiO_2 substrate. Inset: UV-vis spectrum of diluted colloidal paste showing a plasmonic peak at 416 nm.

SERS spectra of Fellini inks taken with Au nanorod nanopastes

In order to clarify the contribution of different factors in the observed SERS spectra, further SERS measurements were taken at 514 nm using Au nanorod nanopastes with LSPR centered at 757 nm. For blue Fellini ink a small difference was observed between spectra taken under NR, SERS_Ag nanopastes and SERS Au nanopastes conditions (see Figure S4a). 1650 cm^{-1} with Ag nanopastes attributed to the presence of a red rhodamine component (and not observable with Au nanopastes) could be an indication of some weak SERS effects taking place in the blue ink. Regarding the green Fellini ink small difference was observed between NR and SERS_Ag nanopastes, resulting in appearance of diagnostic peaks not observable in the NR spectrum (Figure S4b). This suggested that weak SERS effects took place at 514 nm excitation wavelength. On the other hand, the green Fellini SERS spectrum taken at 514 nm with Au nanopaste showed no enhancement and therefore no occurrence of SERS effects. For pink Tombow Fellini no SERS effect with Au nanopastes was observed (see Figure S4c). In these conditions the excitation wavelength is not in plasmonic resonance with Au nanopastes and we did not observe any enhancement in the pink Tombow spectrum, in spite of an absorbance molecular resonance with the identified dye eosin Y. This would suggest that a plasmonic effect occurs with Ag nanopastes responsible for the strong enhancement observed.

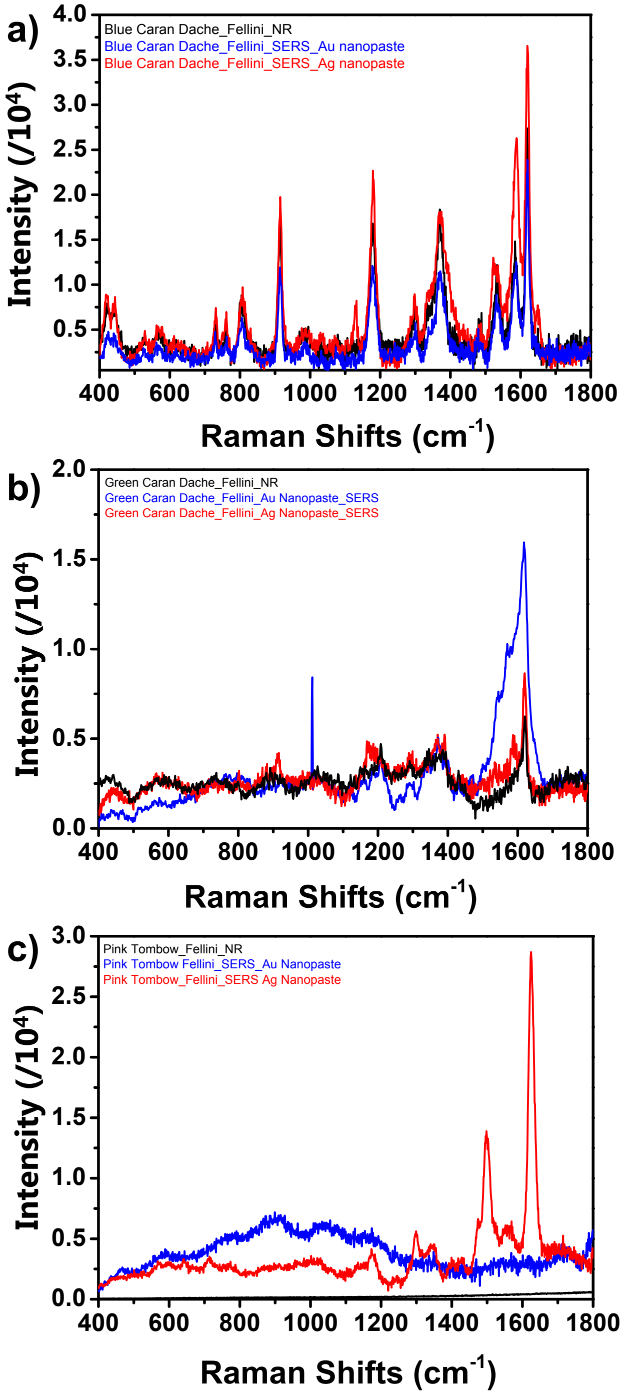

Figure S4. NR, Ag_nanopaste SERS and Au_nanopaste SERS spectra obtained for a) blue Caran D'Ache Fibralo series 100 ink; b) green Caran D'Ache Fibralo series 100 ink and c) pink Tombow ABT 813.

Identification of dyes and peak assignments

Table S1. List of analyzed inks, identified dyes, associated peak positions and peak attribution.

Analyzed Ink	Identified dye	Peak position (cm^{-1})	Attribution
**Pink Tombow ABT 813**	Eosin Y[	1620	xanthene ring C−C stretching
		1570	benzene ring C=C stretching
		1503	xanthene and benzene ring C−C stretching
		1473	xanthene ring C−C stretching
		1414	xanthene ring C−C stretching
		1345	COO sym stretching
		1316	benzene ring and COO sym stretching
		1283	xanthene and benzene ring C−C stretching
		1176	xanthene and benzene ring C−C stretching and COO sym stretching
		1058	benzene ring and COO sym stretching
		1019	xanthene ring breathing and C−Br stretching
		773	xanthene ring stretching
		712	xanthene out of plane ring deformation
		640	xanthene ring stretching and benzene ring stretching
		402	xanthene breathing, benzene ring and COO stretching
**Green/Blue Caran D'Ache Fibralo series 100**	Erioglaucine	1618	aromatic C-C stretching
		1587	aromatic C-C stretching
		1534	aromatic C-C stretching
		1481-1452	ring deformation
		1420-1370	N-C aromatic stretching
		1279	aromatic C-C stretching
		1185	in-plane ring CH bending
		1073	CH bending of CH2CH3
		918	C-C ring stretching

		805	out of plane ring CH bending
		760	SO^{3-} bending
**Blue Caran D'Ache Fibralo series 100**	Crystal Violet	1583-1532	aromatic C-C stretching
		1444	ring deformation
		1372	N-C aromatic stretching
		1300	asymmetric stretching of central C-C-C, bending of the ring C-C-C and C-H
		1177	asymmetric benzene mode, asymmetric stretching C-C-C
		913	C-C ring stretching
		809	asymmetric benzene mode
		439	symmetric benzene mode and bending of C-N-C
		417	asymmetric benzene mode, central C-C-C, bending of C-N-C
	Rhodamine	1650	xanthene ring stretching, in plane C-H bending
		1524	asymmetrical stretching CC phenyl ring
		1395	symmetrical stretching CC in xanthene ring
		1283	C-C stretching xanthene and benzene ring, COC stretching
